# The Influence of Overnight Orthokeratology on Ocular Surface and Meibomian Gland Dysfunction in Teenagers with Myopia

**DOI:** 10.1155/2019/5142628

**Published:** 2019-01-21

**Authors:** Xiu Wang, Jing Li, Rui Zhang, Na Li, Yi Pang, Yan Zhang, Ruihua Wei

**Affiliations:** ^1^Tianjin Medical University Eye Hospital, Tianjin Medical University Eye Institute, College of Optometry and Ophthalmology, Tianjin Medical University, Fukang Rd. 251, Nankai District, Tianjin 300384, China; ^2^Associate Dean for Research, Illinois College of Optometry, 3241 S, Michigan Ave, Chicago, IL 60616, USA

## Abstract

**Purpose:**

The aim of this study was to investigate the effect of overnight orthokeratology (OOK) on ocular surface and meibomian gland dysfunction in teenagers with myopia.

**Methods:**

A total of 59 subjects were recruited in this prospective study. The following tests were performed before and after 1, 3, 6, 12, and 24 months of OOK lens wear, including ocular surface disease index (OSDI) questionnaire, slit-lamp examination, and Keratograph 5M.

**Results:**

No infectious keratitis occurred during the study. OSDI scores increased gradually and reached the maximum at 6 months of OOK wear (*P* < 0.001). The meniscus height was significantly increased at 1 and 3 months after the initiation of OOK (*P*=0.006, *P*=0.035). The corneal fluorescein staining at 1, 3, 6, 12, and 24 months after wearing OOK were all increased than the prewearing level with significant difference (*P*=0.014, *P*=0.036, *P* < 0.001, *P* < 0.001, and *P*=0.008, respectively). The first and the average tear film NIKBUT were all higher than the prewearing level, but there was no significant difference between every follow-up time points (*P* > 0.05). The lid margin abnormalities were significantly increased (*P*=0.003, *P*=0.038, and *P*=0.015) at 6, 12, and 24 months after the initiation of OOK. There was no significant difference in the meibomian gland orifice scores at each follow-up time points compared to the prewearing level (*P* > 0.05). The meibomian gland lipid secretion scores after wearing OOK were higher than those of the prewearing level, however, without statistically significant difference (*P* > 0.05). No significant differences of the degree of difficulty of lipid excretions were detected after the initiation of OOK (*P* > 0.05). There was no significant difference in meibomian gland dropout scores between all follow-up time points and the prewearing level (*P*=1.000).

**Conclusion:**

OOK increased the symptoms of dry eye and decreased the function of tear film by affecting the meniscus height and BUT. OOK did not affect the function of meibomian glands.Clinical Study registration number: ChiCTR18000185708.

## 1. Introduction

Overnight orthokeratology (OOK), an optical compensation mainly for correcting low-to-moderate myopia, is a method to correct refractive errors using custom-designed rigid lenses to temporarily modify the curvature of the cornea [1, 2]. The increased use of OOK is due to its capability to slow down the progression of myopia [3–5]. Recent studies have shown that wearing OOK lenses during nighttime is safe, although they might slightly damage the ocular surface [6, 7]. The effects of OOK on tear film components [8], such as inflammatory mediators [9], have been studied. Several studies have demonstrated that OOK lenses can damage the ocular surface [10–12], and, in serious cases, they can even lead to an infectious corneal ulcer [13].

The purpose of this study was to determine the effects of OOK lens on the ocular surface and meibomain gland over a wearing period of two years in myopic children from 7 to 18 years.

## 2. Materials and Methods

### 2.1. Materials

In this prospective study, 59 myopic subjects (average age 12.03 ± 2.31 years, range 7 to 18 years; male to female ratio is 1 : 1.19) were recruited at Tianjin Medical University Eye Hospital (Tianjin, China) from January to June 2015. Patients with a history of systemic or ocular treatment, contact lens wear, keratitis, ocular allergic disease, any other ocular surface disease, glaucoma, active and chronic uveitis, or previous ocular surgery or injury were excluded. The written informed consent was obtained from the patients' parents. All the procedures and the informed consent form of this study were approved by the Institutional Review Board in Tianjin Medical University Eye Hospital, Tianjin, China. All the procedures performed were in accordance with the tenets of the Declaration of Helsinki.

### 2.2. Information on Lenses

All participants were fitted empirically with the Emerald™ Contact Lens (oprifocon A, Euclid Systems Corporation, USA). The lenses were fitted in accordance with the manufacturer's guidelines. Ideally, the lens should be fitted in the 3 to 6 mm dark area that is not stained with fluorescein. A 1-2 mm wide fluorescein-filling area is located next to the central reverse arc, and the width of the location arc parallel to the cornea is 2 to 3 mm. The periarc has 0.5 to 1 mm fluorescein filling. There should be a 1-2 mm area for the movement of lens during blinking. After a blink-associated movement, the lens should automatically return to the central cornea. At initial dispense, the lenses were evaluated, and the subjects were trained with the insertion, removal, care, and cleaning of their lenses. Even if the patients reported no changes in visual acuity, the lenses were replaced annually. All procedures for the fitting, prescription, and replacement of OOK lenses were performed by a single experienced specialist. The subjects were provided with O_2_ Care Milpha® solution for daily lens cleaning and disinfection and Progent® intensive cleaner for monthly lens cleaning (Menicon Co., Ltd., Nagoya, Japan). The appropriate contact lens fit was accomplished and verified by corneal topography (Orbscan Topography System II; Bausch & Lomb, Salt Lake City, UT). All subjects were asked to sleep uninterruptedly for at least 8 h and remove the lenses immediately upon waking.

### 2.3. Methods

All subjects were examined by the same experienced examiner before and after 1, 3, 6, 12, and 24 months of OOK lens wear. In each visit, the patients' discomforts were assessed by an ocular surface disease index (OSDI) questionnaire. The ocular surface was examined by Keratograph 5M and a slit lamp. At the final visit, at 24 months after wearing OOK, the incidence of complications was recorded.

### 2.4. Questionnaire on Dry Eye

The OSDI is valid and reliable for evaluating the severity of dry eye disease, even the dry eye in children [14, 15]. Each subject was asked to complete an OSDI questionnaire for assessment of ocular surface symptoms and the severity of dry eye.

### 2.5. Slit-Lamp Examinations of the Anterior Segment

The following examinations were carried out sequentially using a slit-lamp: corneal fluorescein staining, lid margin abnormalities, meibomian gland orifices, quality of meibomian gland lipid secretion, and difficulty of lipid excretions.

Corneal fluorescein staining was graded from 0 to 12, which was a sum of the scores of corneal four quadrants. The four quadrants of cornea were carefully examined and scored individually as 0 (no staining), 1 (mild staining with a few scattered dots of stains), 2 (moderate staining between 1 and 3), and 3(severe staining with confluent stains or corneal filaments) [16].

Lid margin abnormalities were scored according to the following 4 signs: vascular engorgement, lid margin irregularity, obstructed meibomian gland orifices, and anterior or posterior displacement of the mucocutaneous junction [17]. The lid margin abnormalities score ranged from 0 to 4.

The quality of meibomian gland orifices was graded semiquantitatively in the central eight glands of the lower right eyelid. Grade 0 is normal, i.e., no obstruction of orifice, and the orifices were covered with a thin and smooth fluid; Grade 1 was obstruction of one or two meibomian gland orifices, or there are secretions or occlusion in one or two meibomian gland orifices; Grade 2 was obstruction of two or three meibomian gland orifices with thick fluid; Grade 3 was obstruction or narrowing of almost half of the meibomian gland orifices; Grade 4 was obstruction or narrowing of more than half of the meibomian gland orifices with sticky secretions.

The quality of meibomian gland lipid secretion was graded semiquantitatively in the central eight glands of the lower right eyelid [18]. Grade 0, clear fluid; Grade 1, cloudy fluid; Grade 2, cloudy, particulate fluid; and Grade 3, inspissated, toothpaste-like fluid.

Difficulty of lipid excretions was graded by squeezing central meibomian gland of the lower eyelid and evaluating the degree of the secretion discharge in the central 5 glands. Grade 0: five glands had secretions out; Grade 1: three to four glands had secretions out; Grade 2: one to two glands had secretions out; Grade 3: no glands had secretions out.

### 2.6. Keratograph® 5M: Noninvasive Measurement for Ocular Surface

Keratograph® 5M inspection items included noninvasive keratographic tear film break-up time (the first keratographic break-up time and the average keratographic break-up time), noninvasive tear meniscus height, and meibography. The tests were conducted first on the right eye and then on the left. Three measurements were recorded. Keratograph® 5M was used to grade the eyelid using the degree of meibomian gland dropout as meiboscore [17]: Grade 0: no loss of meibomian gland; Grade 1: loss of <1/3 of the whole gland area; Grade 2: loss of 1/3–2/3 of the whole gland area; and Grade 3: loss of >2/3 of the whole gland area. The meiboscore of each eye was calculated as the sum of the scores from both upper and lower eyelids.

### 2.7. Statistical Analysis

Statistical analyses were performed using Statistical Program for Social Sciences 19.0 (IBM SPSS Inc., New York, NY, USA). No statistically significant difference was found in all parameters between the right and left eyes. Thus, only the data on the right eyes were used for further analyses. All data were expressed as mean ± standard deviation and tested by D'Agostino and Pearson omnibus normality tests. The data with Gaussian distribution were tested by the Levene test to confirm the homogeneity of variance. The data collected before and after the wearing of OOK lenses were analyzed by two-way ANOVA followed by Tukey's post hoc test. The data with nonparametric distribution were analyzed by the Wilcoxon rank-sum test. *P* values less than 0.05 were considered significant.

## 3. Results

The mean spherical equivalent of the subjects was −3.70 ± 1.39 diopter (D). The baseline ocular parameters and subsequent changes at every follow-up time point are presented in Table 1. Allergic conjunctivitis occurred in six subjects. They were instructed to temporarily stop wearing OOK lenses and use 0.1% olopatadine eye drops (Alcon Laboratories, Inc.) at b.i.d. One month after the allergic conjunctivitis was resolved, the subjects resumed OOK lenses. No infectious keratitis was observed during the study period. No subject dropped out during the study.

**Figure 1:**
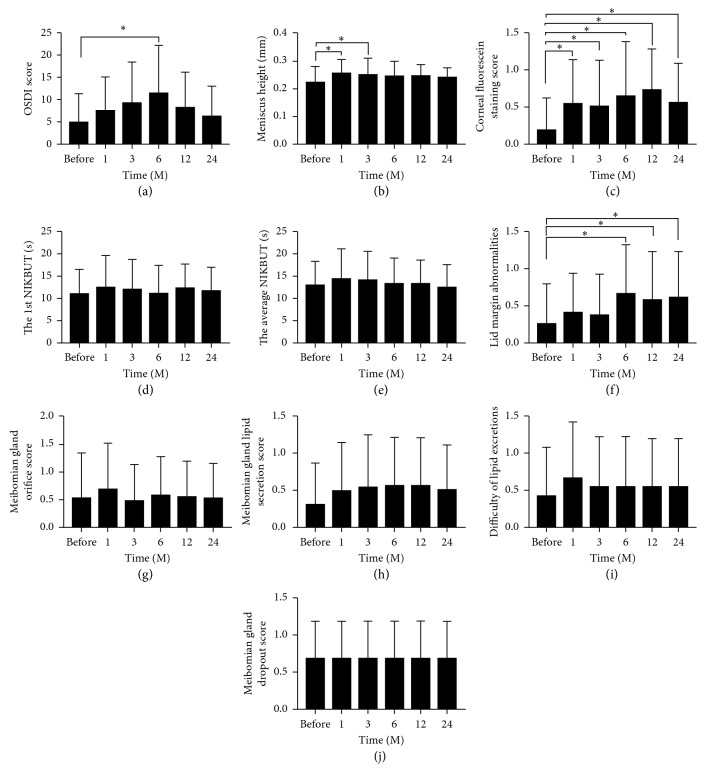
Ocular surface parameters after wearing of OOK. The parameters, including OSDI score (a), meniscus height (b), corneal fluorescein staining score (c), the first keratographic tear film BUT (d), the average keratographic tear film BUT (e), lid margin abnormalities (f), meibomian gland orifice score (g), meibomian gland lipid secretion score (h), difficulty of lipid excretions (i), and meibomian gland dropout scores (j) were compared before and at follow-up time points after wearing OOK. ^*∗*^*P* < 0.05 when compared with prewearing OOK.

Compared with the baseline values, OSDI scores for ocular discomfort increased with the wearing of OOK and peaked at the 6-month visit (Figure 1(a), *P* < 0.001, for 6 months vs baseline). OSDI scores began to decline at 12 and 24 months after wearing OOK and had no significant difference with the baseline values (*P*=0.275, for 12 months vs baseline; *P*=0.947, for 24 months vs baseline).

The meniscus height was significantly increased at 1 and 3 months after the initiation of OOK (Figure 1(b), *P*=0.006, for 1 month vs baseline; *P*=0.035, for 3 months vs baseline). However, no significant differences were detected between the meniscus heights at 6, 12, and 24 months and that at the baseline (*P*=0.190, for 6 months vs baseline; *P*=0.117, for 12 months vs baseline; *P*=0.392, for 24 months vs baseline).

The corneal fluorescein staining at 1, 3, 6, 12, and 24 months after wearing OOK were all significantly increased in comparison to the prewearing level (Figure 1(c), all *P* < 0.05, when vs baseline). However, there was no significant difference among follow-up time points (all *P* > 0.05). Despite the increments in the corneal fluorescein staining, the subjects opted to continue wearing OOK under careful monitoring.

The first tear film keratographic BUT and the average tear film keratographic BUT were higher than the prewearing level, but there was no significant difference among every follow-up time points (Figures 1(d) and 1(e), all *P* > 0.05).

The lid margin abnormalities were significantly exacerbated at 6, 12, and 24 months after the initiation of OOK (Figure 1(f), *P*=0.003, 6 months vs baseline; *P*=0.038, 12 months vs baseline; *P*=0.015, 24 months vs baseline). However, no significant difference was detected between the levels at 1 and 3 months and that at the baseline (Figure 1(f), *P*=0.726, 1 month vs baseline; *P*=0.885, 3 months vs baseline).

There was no significant difference in meibomian gland orifice scores at each follow-up time points compared with the prewearing level (Figure 1(g), all *P* > 0.05). meibomian gland orifice scores showed a trendy increase at 1-month after OOK wear with no statistical significance. The meibomian gland secretion scores after wearing OOK were higher than the prewearing score but without statistical significance (Figure 1(h), all *P* > 0.05). There was no significant difference between every follow-up time points (all *P* > 0.05). No significant differences in the degree of lipid excretion were detected among all the time points prior to and after the OOK wearing (Figure 1(i), all *P* > 0.05).

There were no significant differences in meibomian gland dropout scores between all the follow-up time points and the prewearing level (Figure 1(j), all *P* > 0.05).

## 4. Discussion

Due to the poor coordination of young children, the data reported in this field are limited and often insufficient to evaluate the effect of overnight orthokeratology on ocular surface and meibomian gland dysfunction in the young individuals. In this study, we employed 6 subjective parameters on the functions of ocular surface and meibomian gland, including OSDI questionnaire, corneal fluorescein staining, lid margin abnormalities, meibomian gland orifices, quality of meibomian gland lipid secretion, and difficulty of lipid excretions, as well as 4 objective parameters, including the first keratographic break-up time, the average keratographic break-up time, tear meniscus height, and meibography. The 10 ocular surface parameters in total make the results of the examinations more comprehensive. More importantly, the 4 objective parameters were measured by the noninvasive Keratograph 5M, which to a certain extent overcomes the difficulty in coordination from the young patients and renders the results of our study more precise.

To date, the safety of using orthokeratology has acquired increasing attention. Infectious keratitis has been reported in patients wearing OOK in both case reports and clinical studies [19–21]. However, no infectious keratitis was detected in this study.

Contact lens can cause eye discomfort (CLD). Several factors, including increased evaporation, thinning of tear film, and incomplete blink, have been proposed as the potential causes of CLD [22, 23]. However, OOK does not involve open-eye lens wear, thus influence resulting from evaporation, tear film thinning, and partial blinking may be minimal compared with conventional contact lens wear. OSDI is an indicator of dry eye and OOK subjective symptoms. In our study, a few of the subjects temporarily stopped wearing OOK due to eye discomfort. Forty out of 59 subjects exhibited increased OSDI scores after wearing OOK; however, the scores declined after 6 months, indicating an improved tolerance to OOK with an extension of wearing.

Meniscus height after wearing OOK significantly increased compared with that at the prewearing level, indicating that OOK wearing results in an increase in tear secretion. Several studies suggested that OOK, as an eye foreign body, can stimulate excessive tearing [24, 25]. Carracedo's study showed that wearing OOK for 1 month caused no considerable changes in tear function and did not lead to tear-reduction-related symptoms such as dry eye [26]. In this study, after wearing OOK for a month, the OSDI was not significantly different from that at the prewearing level. Moreover, the first tear film keratographic BUT and the average tear film keratographic BUT did not substantially differ among all the follow-up time points.

Although the score of corneal fluorescein staining after wearing OOK was higher than that at the baseline, there was no further increase in the corneal fluorescein staining during the follow-ups. The OOK lenses are composed of rigid and gas-permeable materials with high oxygen permeability. However, long-term wearing still can cause hypoxia to the cornea. Corneal fluorescein staining is the most common complication of OOK lens use [27, 28]. In the study conducted by Li et al., corneal epithelial staining increased after lens wearing with most of the patients graded as I staining and no patient graded more than II [29]. The authors stated that the effect of OOK on the corneal epithelium was minor and reversible [29]. Furthermore, Chan et al. have reported that corneal staining is the most commonly observed complication with OOK; they also suggest that this complication is due to thinning of the central corneal epithelium, improper lens fitting, corneal hypoxia, hypersensitivity to contact lens solution, mechanical abrasion caused by the build-up of deposits on the lens' back surface, lens binding, and incorrect removal of a bound lens in the morning [27]. In our study, subjects were not given artificial tears to prevent corneal staining but were closely monitored for possible changes in the ocular surface. Corneal fluorescence staining score at each follow-up time point was higher than that at the prewearing level, and most patients were at grade I, whereas only 10.52% of the patients reached grade II. No subject's score exceeded grade II.

The indicators of meibomian gland dysfunction (MGD) are lid margin abnormalities, meibomian gland orifice scores, meibomian gland lipid secretion scores, difficulty of lipid excretions, and meibomian gland dropout scores. MGD has recently been considered as a major pathogenic factor for the development of evaporative dry eye, even in children [30]. Loss of meibomian glands is also deemed as a potential cause of CLD [18]. In our study, the meibomian gland dropout maintained its stability after 24 months of wearing OOK, which indicates no observable effect of OOK on the meibomian gland structure. In a previous study, two of the 58 patients with meibomian gland distortion exhibited modification before wearing OOK [12]. Distortion occurred in the first stage of morphologic changes of meibomian glands [31]. Future studies are needed in order to identify subtle meibomian gland changes. Six of the 59 subjects in this study experienced allergic conjunctivitis. The prevalence of allergic conjunctivitis is higher in children compared with adults [32] Numerous children with this condition also suffer from dry eye which complicates the diagnosis of ocular surface disease [33]. In Arita et al.'s study, allergic conjunctivitis is associated with increased meibomian gland duct distortion [34]. Our subjects may have developed subclinical allergic conjunctivitis and distorted meibomian glands at the beginning of the study, and OOK merely aggravated the preexisting allergic conjunctivitis and caused further distortion or loss of the meibomian gland [12].

Our study has limitations. First, this work did not include a control group comprising nonOOK wearers. The ocular surface parameters of postwearing OOK were compared to those of prewearing OOK, instead of those in the nonwearing control subjects. The ocular surface parameters in the nonwearing control subjects may change within the two-year study period. However, this possibility is small when considering the age of the subjects (7 to 18 years old) and the low incidence of dry eye in children. Another limitation is that certain data, such as the parameters on ocular surface and meibomian gland, were subjective. Thus, further studies are warranted to clarify the reversibility of these changes.

## Figures and Tables

**Table 1:** The baseline ocular parameters and subsequent changes in the values at 1, 3, 6, 12, and 24 months after the wearing of OOK.

	Baseline	1 month	3 months	6 months	12 months	24 months
OSDI score	4.81 ± 6.64	7.48 ± 7.74	9.16 ± 9.43	11.40 ± 11.01	8.13 ± 8.19	6.21 ± 6.95
Meniscus heights	0.22 ± 0.06	0.25 ± 0.05	0.25 ± 0.06	0.24 ± 0.06	0.24 ± 0.04	0.24 ± 0.04
Corneal fluorescein staining scores	0.19 ± 0.43	0.54 ± 0.60	0.51 ± 0.63	0.64 ± 0.74	0.73 ± 0.55	0.56 ± 0.53
The 1st keratographic BUT	11.03 ± 5.53	12.49 ± 7.20	11.99 ± 6.84	11.10 ± 6.35	12.37 ± 5.41	11.73 ± 5.33
The average keratographic BUT	13.02 ± 5.39	14.41 ± 6.77	14.16 ± 6.48	13.31 ± 5.81	13.33 ± 5.33	12.49 ± 5.11
Lid margin abnormalities	0.25 ± 0.54	0.41 ± 0.53	0.37 ± 0.55	0.66 ± 0.66	0.58 ± 0.65	0.61 ± 0.61
Meibomian gland orifice scores	0.53 ± 0.82	0.68 ± 0.84	0.47 ± 0.65	0.58 ± 0.70	0.54 ± 0.65	0.53 ± 0.63
Meibomian gland lipid secretion scores	0.31 ± 0.56	0.49 ± 0.65	0.54 ± 0.70	0.56 ± 0.65	0.56 ± 0.65	0.51 ± 0.59
Difficulty of lipid excretions	0.42 ± 0.65	0.66 ± 0.76	0.54 ± 0.68	0.54 ± 0.68	0.54 ± 0.65	0.54 ± 0.65
Meibomian gland dropout scores	0.68 ± 0.51	0.68 ± 0.51	0.68 ± 0.51	0.68 ± 0.51	0.68 ± 0.51	0.68 ± 0.51

## Data Availability

The data used to support the findings of this study are available from the corresponding author upon request.
